# HTLV-1 Propels Thymic Human T Cell Development in “Human Immune System” Rag2^-/-^ gamma c^-/-^ Mice

**DOI:** 10.1371/journal.ppat.1002231

**Published:** 2011-09-01

**Authors:** Julien Villaudy, Mélanie Wencker, Nicolas Gadot, Nicolas A. Gillet, Jean-Yves Scoazec, Louis Gazzolo, Markus G. Manz, Charles R. M. Bangham, Madeleine Duc Dodon

**Affiliations:** 1 Virologie Humaine, INSERM-U758, Lyon, France; 2 Ecole Normale Supérieure, Lyon, France; 3 UMS3444 BioSciences Lyon-Gerland, Lyon, France; 4 Cancer Research UK, London Research Institute and King's College, London, United Kingdom; 5 Anipath, UFR Médecine Lyon-RTH Laennec, Lyon, France; 6 Department of Immunology, Wright-Fleming Institute, Imperial College London, London, United Kingdom; 7 Molecular and Cellular Epigenetics, Interdisciplinary Cluster for Applied Genoproteomics (GIGA) of University of Liège (ULg), Liège, Belgium; 8 Institute for Research in Biomedicine (IRB), Bellinzona, Switzerland; 9 University and University Hospital Zürich, Division of Hematology, Zürich, Switzerland; University of Pennsylvania School of Medicine, United States of America

## Abstract

Alteration of early haematopoietic development is thought to be responsible for the onset of immature leukemias and lymphomas. We have previously demonstrated that Tax_HTLV-1_ interferes with ß-selection, an important checkpoint of early thymopoiesis, indicating that human T-cell leukemia virus type 1 (HTLV-1) infection has the potential to perturb thymic human αβ T-cell development. To verify that inference and to clarify the impact of HTLV-1 infection on human T-cell development, we investigated the *in vivo* effects of HTLV-1 infection in a “Human Immune System” (HIS) Rag2^-/-^γ_c_^-/-^ mouse model. These mice were infected with HTLV-1, at a time when the three main subpopulations of human thymocytes have been detected. In all but two inoculated mice, the HTLV-1 provirus was found integrated in thymocytes; the proviral load increased with the length of the infection period. In the HTLV-1-infected mice we observed alterations in human T-cell development, the extent of which correlated with the proviral load. Thus, in the thymus of HTLV-1-infected HIS Rag2^-/-^γc^-/-^ mice, mature single-positive (SP) CD4^+^ and CD8^+^ cells were most numerous, at the expense of immature and double-positive (DP) thymocytes. These SP cells also accumulated in the spleen. Human lymphocytes from thymus and spleen were activated, as shown by the expression of CD25: this activation was correlated with the presence of *tax* mRNA and with increased expression of NF-kB dependent genes such as *bfl-1*, an anti-apoptotic gene, in thymocytes. Finally, hepato-splenomegaly, lymphadenopathy and lymphoma/thymoma, in which Tax was detected, were observed in HTLV-1-infected mice, several months after HTLV-1 infection. These results demonstrate the potential of the HIS Rag2^-/-^γ_c_^-/-^ animal model to elucidate the initial steps of the leukemogenic process induced by HTLV-1.

## Author Summary

Human T-cell leukemia virus type 1 (HTLV-1) is a retrovirus responsible for the development of Adult T cell Leukemia, an aggressive malignant lymphoproliferation that occurs several decades after viral exposure in some individuals infected when neonates *via* breastfeeding. Here, we used mice that were humanized by transplantation of human hematopoietic stem cells isolated from cord blood. Such an animal model may reproduce key aspects of *in vivo* infection with HTLV-1 and improve the understanding of the early steps of the leukemogenic process. These humanized mice when inoculated with HTLV-1 were indeed productively infected, as shown by the detection and the expression of the provirus in human cells. We observed that T-cell development in the thymus of these mice was profoundly altered, as ascertained by the increased proliferation of activated mature T lymphocytes that accumulated in the thymus as well as in the spleen. These alterations of human thymic T-cell development may be linked to the development of ATL–like pathologies. These results show that this humanized mouse model provides an invaluable tool not only to study the early stages of the infection leading to the development of malignant lymphoproliferations, but also to design new therapeutic approaches.

## Introduction

HTLV-1 (Human T cell Leukaemia Virus, type 1), the only retrovirus associated with a neoplastic disease in humans, is the etiologic agent of Adult T-cell Leukaemia (ATL), an aggressive clonal lymphoproliferative disorder of mature CD4^+^ CD25^+^ T cells [1]-[3]. It is considered that HTLV-1 infection early in life predisposes to ATL. Indeed, epidemiological studies have stressed that ATL develops after an extremely long (several decades) latency period, often in patients who were infected as neonates through breastfeeding [4]. T-cell leukemias such as T-ALL (T-cell acute lymphoblastic leukaemia) which mainly occur in children and adolescents, are thought to result from malignant thymocytes arising at defined stages of intrathymic T-cell development [5], [6]. Thus, HTLV-1 infection within the thymus might be an essential event in the pathogenesis of ATL. *In vitro*, HTLV-1 can productively infect human hematopoietic CD34^+^ progenitor cells as well as human immature thymocytes [7]–[9]. It has also been demonstrated that reconstitution of T lymphopoiesis with HTLV-1-infected CD34^+^ cells in severe combined immunodeficient (SCID) mice engrafted with human thymus and liver tissues resulted in the alteration of thymopoiesis, as shown by abnormalities in the size of thymocyte subpopulations [7]. Recently, Banerjee *et al.* have generated a humanized model, which partially recapitulates HTLV-1-induced T-cell leukemogenesis by inoculating immunodeficient mice with human CD34^+^ hematopoietic progenitor cells infected *ex vivo* with HTLV-1 [10].

HTLV-1 is a complex retrovirus that relies upon multiple gene products to accomplish replication and disease. Among them, Tax and HBZ are key regulatory proteins. HBZ, encoded by the antisense strand of the HTLV-1 proviral DNA, is permanently expressed and required for efficient viral infectivity and persistence, whereas Tax has been shown to deregulate a number of cellular genes leading to cellular proliferation, to induce genetic instability, thus contributing to malignant progression [3]. Studies on Tax-transgenic mice that express *tax* under the control of either the HTLV-1 LTR or alternative promoters that target Tax expression to lymphocytes have demonstrated that Tax can perturb lymphocyte functions. The development of tumors in most of these transgenic mouse models also provided evidence of the leukemogenic potential of Tax. Interestingly, when the transgene expression was placed under the control of the Lck promoter, which restricts Tax expression to developing thymocytes, an ATL-like phenotype developed in the transgenic mice [11]. These observations suggest that the leukemogenic activity of Tax is related to T-cell development in the thymus. We have previously reported that Tax, by silencing E2A transcription factors, down-regulates the expression of the pTα gene thus perturbing β-selection, the early and critical checkpoint of human T-cell development in the thymus [12], [13]. These *in vivo* and *in vitro* studies indicated that HTLV-1 infection in the thymus might be a prerequisite for the malignant lymphoproliferation associated with this retroviral infection.

In the present study, we first investigated the *in vivo* effects of HTLV-1 infection on human T-cell development in “Human Immune System” (HIS) mice by transplanting human CD34^+^ cord blood cells into conditioned newborn BALB/c Rag2^-/-^γ_c_^-/-^ recipient mice. The resulting HIS mice were infected with HTLV-1, at a time when the successive stages of the αβT-cell development are evident in the thymus of these mice. In the HTLV-1-infected Rag2^-/-^γ_c_^-/-^ mice, we observed efficient viral expression and profound alterations in human thymopoiesis, as shown by the accumulation of activated mature single-positive CD4^+^ and CD8^+^ lymphocytes in the thymus and in the spleen. The role of Tax in that process is indicated by an increased expression of NF-κB-dependent genes such as CD25 and Bfl-1 in thymocytes of these mice. In addition, abnormalities frequently observed in ATL such as hepatosplenomegaly, lymphadenopathy and lymphoma were detected in HTLV-1-infected mice, several months after HTLV-1 infection. This HIS Rag2^-/-^γ_c_^-/-^ animal model might be of great interest to decipher the initial steps of the leukemogenic process induced by HTLV-1.

## Results

**Figure 1:**
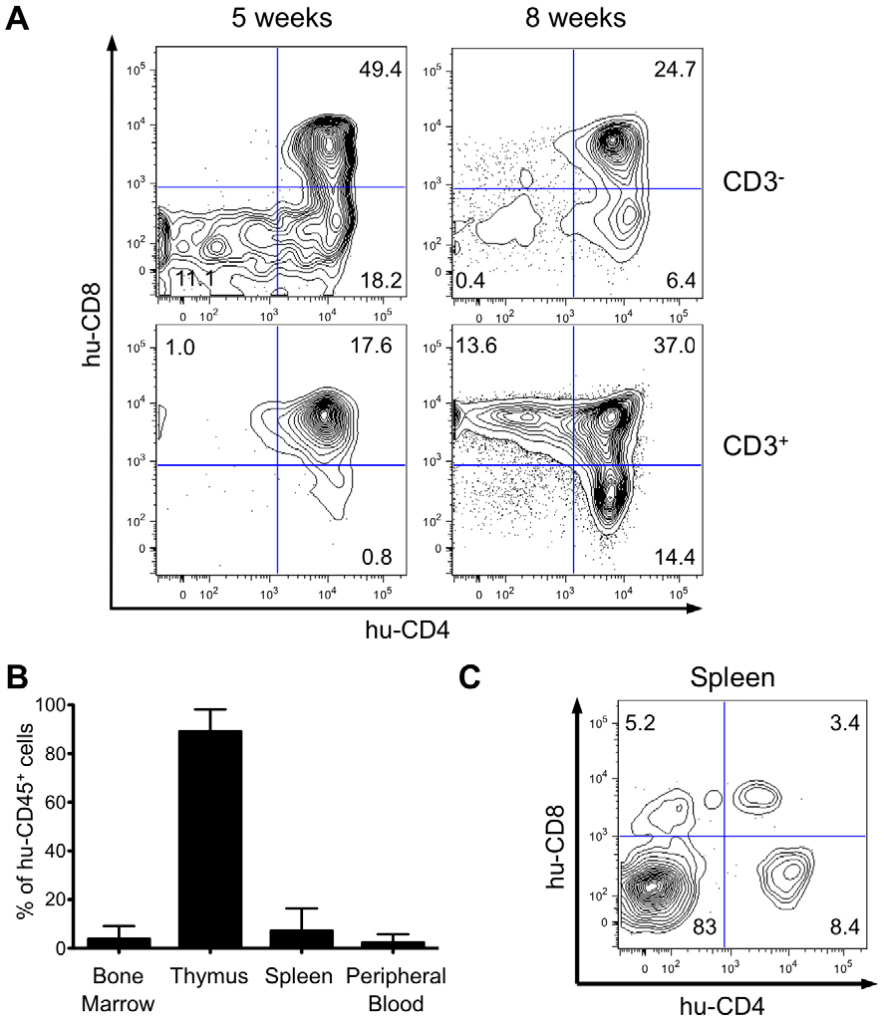
Human hematopoietic cell engraftment and T-cell development in HIS Rag2^-/-^γ_c_^-/-^ mice. (**A**) Efficient intrathymic *de novo* development of human T-cells in BALB/c Rag2^-/-^γ_c_^-/-^ mice. Animals were sacrificed at 5 and 8 weeks after CD34^+^ cell transplantation. The flow cytometry dot plots show representative examples of staining for human CD3, CD4 and CD8 markers (values on the dot plots are for the frequency of the corresponding populations). (**B**) Frequency of human CD45^+^ cells in bone marrow, spleen, thymus and peripheral blood in HIS Rag2^-/-^γ_c_^-/-^ mice, at 10 to 30 weeks post transplantation. (**C**) Representative flow cytometry analysis of total splenocytes in transplanted mice sacrificed at 7 weeks after transplantation stained for human CD4 and CD8 lymphoid cell surface markers.

HIS Rag2^-/-^γ_c_^-/-^ mice were generated by intrahepatic transplantation of human CD34^+^ cord blood cells into sublethally irradiated immunocompromised newborn BALB/c Rag2^-/-^γ_c_^-/-^ mice [14]-[16]. We first analyzed by flow cytometry the expression of CD4 and CD8 on human CD45^+^ (hu-CD45) cells in the thymus of HIS Rag2^-/-^γ_c_^-/-^ mice transplanted with CD34^+^ cells. Five weeks after transplantation, immature CD3^-^ thymocytes, including the double-negative (DN) CD4^-^CD8^-^ cells, the CD4^+^ immature single-positive (CD4ISP) cells and the double-positive (DP) CD4^+^CD8^+^cells were found to be predominant, whereas a low percentage of mature CD3^+^ T-cells containing DP cells and single-positive (SP) CD4^+^ or CD8^+^ cells was detected. pTα has been shown to be highly expressed in immature thymocytes and more specially in CD4ISP cells, just before the pre-T-cell Receptor complex formation [17], [18]. Eight weeks after transplantation, the population of mature thymocytes predominated over that of immature thymocytes, indicating that human αβT-cell development was completed in the thymus of transplanted HIS Rag2^-/-^γ_c_^-/-^ mice (Figure 1A). At that time, approximately 90% of the cells present in the thymus of the HIS Rag2^-/-^γ_c_^-/-^ mice expressed hu-CD45^+^. Circulating human mature T cells were also detected in the blood and secondary lymphoid organs (Figure 1B). Thus, mature T cells represented about 17% of the hu-CD45^+^ cells in the spleen, 10 weeks after CD34^+^ transplantation (Figure 1C). These observations are in line with those previously published [16] and confirm that the successive maturational steps of human αβT-cell development were completed in the thymus of these mice within two months following human CD34^+^ inoculation.

### Evaluation of proviral load in HTLV-1-infected HIS Rag2^-/-^γ_c_^-/-^ mice

**Figure 2:**
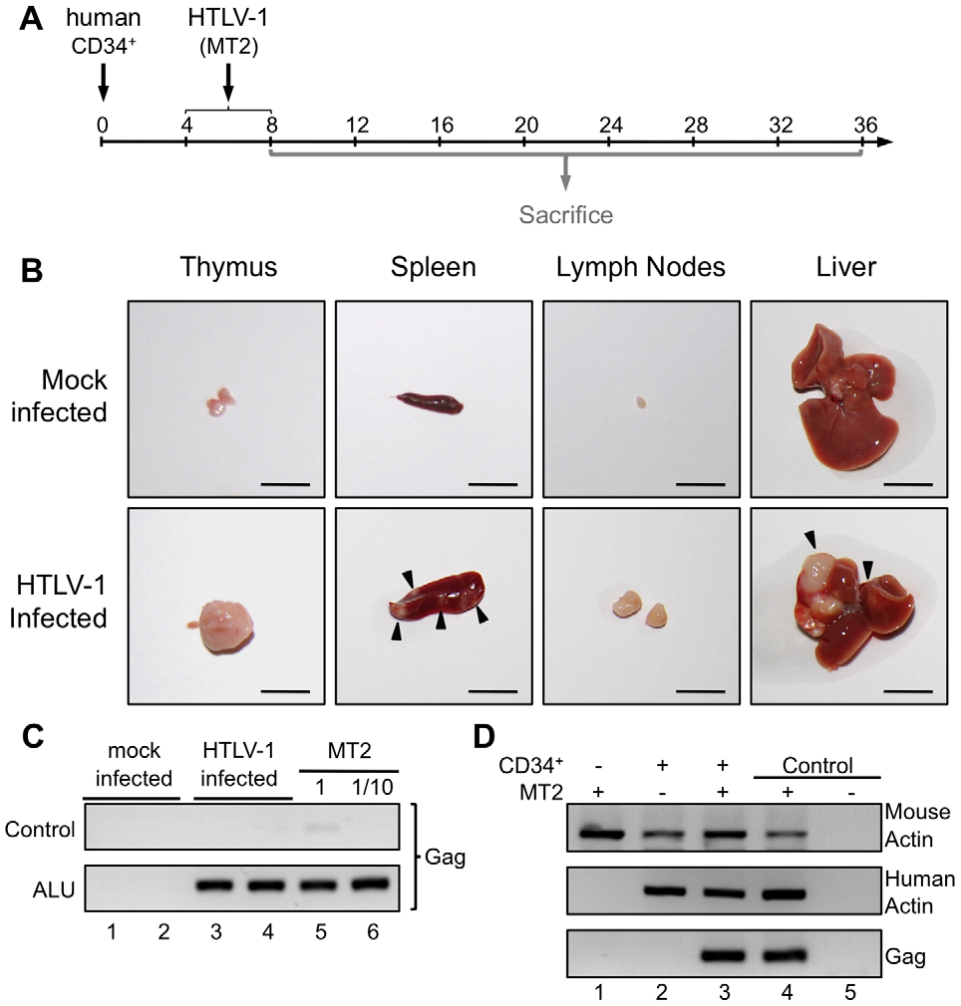
Integration of the HTLV-1 provirus in the genome of human cells in HTLV-1-infected HIS Rag2^-/-^γ_c_^-/-^ mice. (**A**) Schematic representation of the experimental infection set-up. Four to eight weeks after CD34^+^ cell transplantation, the animals were intraperitoneally inoculated with 2×10^6^ lethally irradiated MT2 cells in 100 µl PBS or with PBS alone for the mock-infected mice. Animals were sacrificed and analyzed between 8 and 36 weeks after CD34^+^ cell transplantation. (**B**) Presence of disseminated tumors in HTLV-1 infected HIS Rag2^-/-^γ_c_^-/-^ mice, sacrificed between 16 and 35 weeks after infection and referred to as high proviral load (PVL) mice. Infection resulted in thymoma, and splenomegaly and HTLV-1 positive lymphomas in spleen, in mesenteric lymph nodes or in liver (arrows). Are also shown representative photographs of thymus, spleen, lymph node and liver of 34-week-old HIS Rag2^-/-^γ_c_^-/-^ control mice. Bars, 10 mm (**C**) ALU-PCR was carried out on the DNA extracted from the thymus of mock-infected (lanes 1 and 2) and infected (lanes 3 and 4) mice. The lower panel (two-rounds of PCR, the first one with ALU primers and the second with gag primers) indicates that the HTLV-1 *gag* gene is integrated in the human genome. The upper panel shows the result of control PCR for *gag* carried out on the initial diluted samples without any ALU-PCR. (**D**) HTLV-1 integration does not occur in the mouse genome: PCR for mouse and human actin and for *gag* were performed with the DNA extracted from the spleen of Rag2^-/-^γ_c_^-/-^ mice (not inoculated with human CD34^+^ cells), infected with HTLV-1 (lane 1), or HIS Rag2^-/-^γ_c_^-/-^ mice either mock-infected (lane 2) or infected with HTLV-1 (lane 3).

Taking into account the chronology of the different maturational stages of human αβT-cell development in the thymus of HIS Rag2^-/-^γ_c_^-/-^ mice, HTLV-1 infection of these mice was carried out between 4 and 8 weeks after CD34^+^ transplantation. The mice were inoculated intraperitoneally either with 2×10^6^ lethally irradiated HTLV-1-producing MT2 cells in Phosphate Buffered Saline (PBS) or with PBS only (mock-infected) (Figure 2A). In parallel, untransplanted BALB/c Rag2^-/-^γ_c_^-/-^ mice were inoculated with lethally irradiated MT2 cells.

Autopsy examination between 10 and 35 weeks after HTLV-1 inoculation revealed a significant increase of the size of the thymus, the spleen and lymph nodes of HIS Rag2^-/-^γ_c_^-/-^ mice inoculated with MT2 cells, when compared to those of mock-infected animals. Furthermore tumor-like nodules were frequently observed in the spleen and the liver of MT2-inoculated mice (Figure 2B).

To ascertain that the MT2-inoculated HIS Rag2^-/-^γ_c_^-/-^ mice were indeed infected, we used ALU-PCR to amplify *gag*_HTLV-1_ sequences from human DNA isolated from splenocytes. The results showed that all but two MT2-inoculated HIS Rag2^-/-^γ_c_^-/-^ mice were HTLV-1-infected (Figure 2C, lanes 3, 4 and Figure 2D, lane 3). As expected, viral sequences were not observed in mock-infected HIS Rag2^-/-^γ_c_^-/-^ mice (Figure 2C lanes 1, 2 and Figure 2D, lane 2). Likewise, they were not detected in MT2-inoculated BALB/c Rag2^-/-^γ_c_^-/-^ mice, thus excluding the possibility that the inoculated irradiated MT2 cells were still present in these mice (Figure 2D, lane 1).

**Figure 3:**
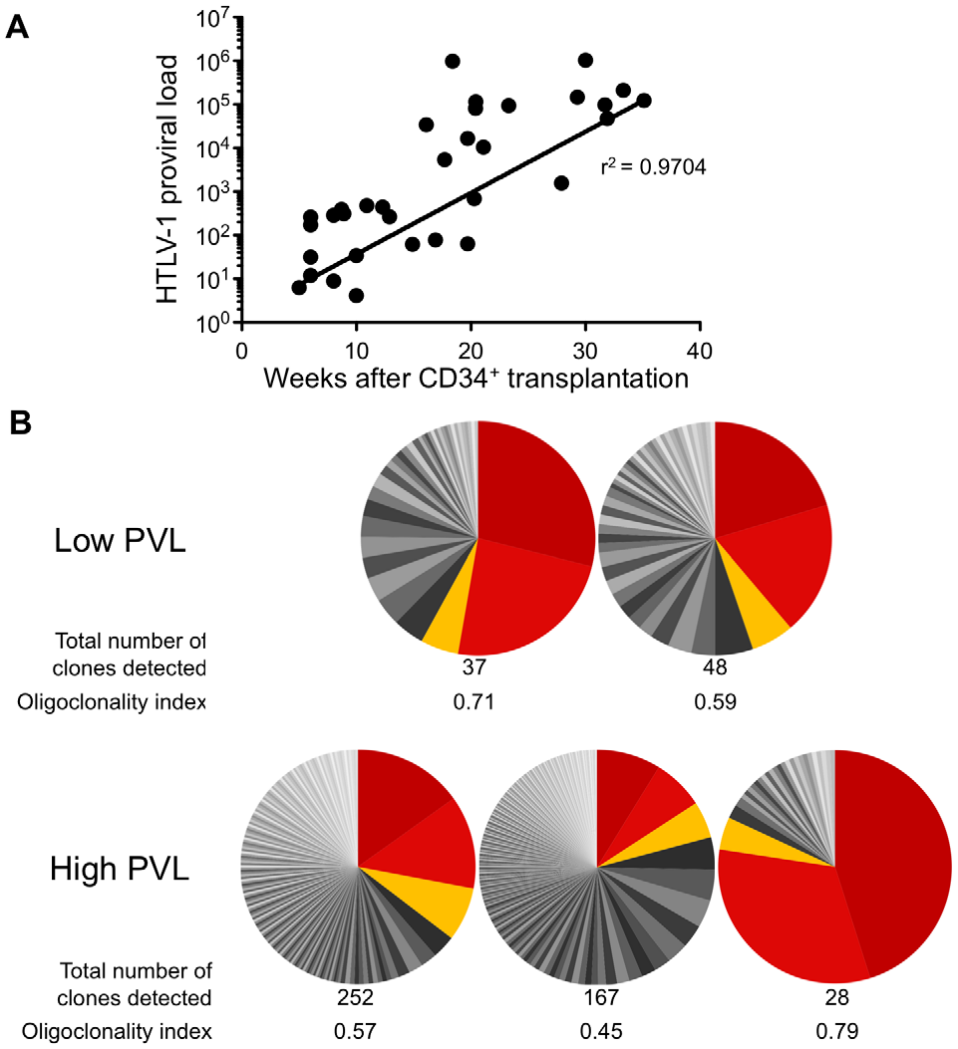
Proviral load and clonality in HTLV-1-infected HIS Rag2^-/-^γ_c_^-/-^ mice. (**A**) Temporal evolution of proviral load (PVL) in the thymus. The HTLV-1 PVL (representing the copy number of *tax* per 10^5^ human cells) were determined by quantitative real-time PCR at the indicated times after CD34^+^ cell transplantation. The PVL level correlates with the length of the infection period (r^2^ = 0.9704). Samples were analyzed at least in duplicate; on all graphs, one dot represents one individual HIS Rag2^-/-^γ_c_^-/-^ mouse. (**B**) Clone frequency distribution of HTLV-1 infected cells in the spleen from 5 different mice. The HTLV-1 clonal structure in each genomic DNA sample is depicted by a pie chart. Each slice represents one unique insertion site (clone); the size of the slice is proportional to the relative abundance of that clone. The 3 most abundant clones were colored in red/orange in each spleen sample. The total number of detected clones was given together with the oligoclonality index values calculated as described in [20].

The HTLV-1 proviral load (PVL) in HTLV-1-infected HIS Rag2^-/-^γ_c_^-/-^ mice was then evaluated by quantitative PCR (qPCR). An increase in the PVL was observed, which correlated with the length of the infection period (Figure 3A). Indeed, a PVL value in the range of 10 to 10^3^ copies per 10^5^ human cells was found in 20 mice that were sacrificed between 5 and 20 weeks after inoculation of MT2 cells. These mice were subsequently referred to as low-PVL mice. Fifteen mice that were sacrificed between 16 and 35 weeks had a PVL between 10^3^ to 10^6^ copies per 10^5^ cells; these were referred to as high-PVL mice. The low PVL values (median 8.9×10^2^ copies/10^5^ cells) were similar to those in healthy human HTLV-1 carriers, whereas the high PVL values (median 1.1×10^5^ copies/10^5^ cells) were similar to those in ATL cells [19]. These results show that HTLV-1 infection of human immune cells was established in the HIS Rag2^-/-^γ_c_^-/-^ mice and that the PVL correlated with the length of the infection period (r^2^ = 0.9704).

We then quantified cellular clonality in the spleen of five representative HTLV-1 infected HIS Rag2^-/-^γ_c_^-/-^ mice, by measuring the frequency of the different HTLV-1 infected clones present in that organ. The calculation of the oligoclonality index is shown in Figure 3B. Each slice in the pie chart represents a single Unique Insertion Site (UIS) and the size of the slice is proportional to the relative abundance of that UIS. In the mouse with the lowest oligoclonality index (0.45), the slices of the pie chart are of similar size. In contrast, in the two mice with the highest clonality index (0.71 and 0.79), two clones were found to predominate. Although there was no significant correlation between the oligoclonality index and the proviral load, these data indicate that oligoclonal proliferation of infected cells occurred in these mice, as observed in naturally infected patients [20].

### The αβT-cell development in HTLV-1 infected HIS Rag2^-/-^γ_c_^-/-^ mice is altered in a PVL-dependent manner

**Figure 4:**
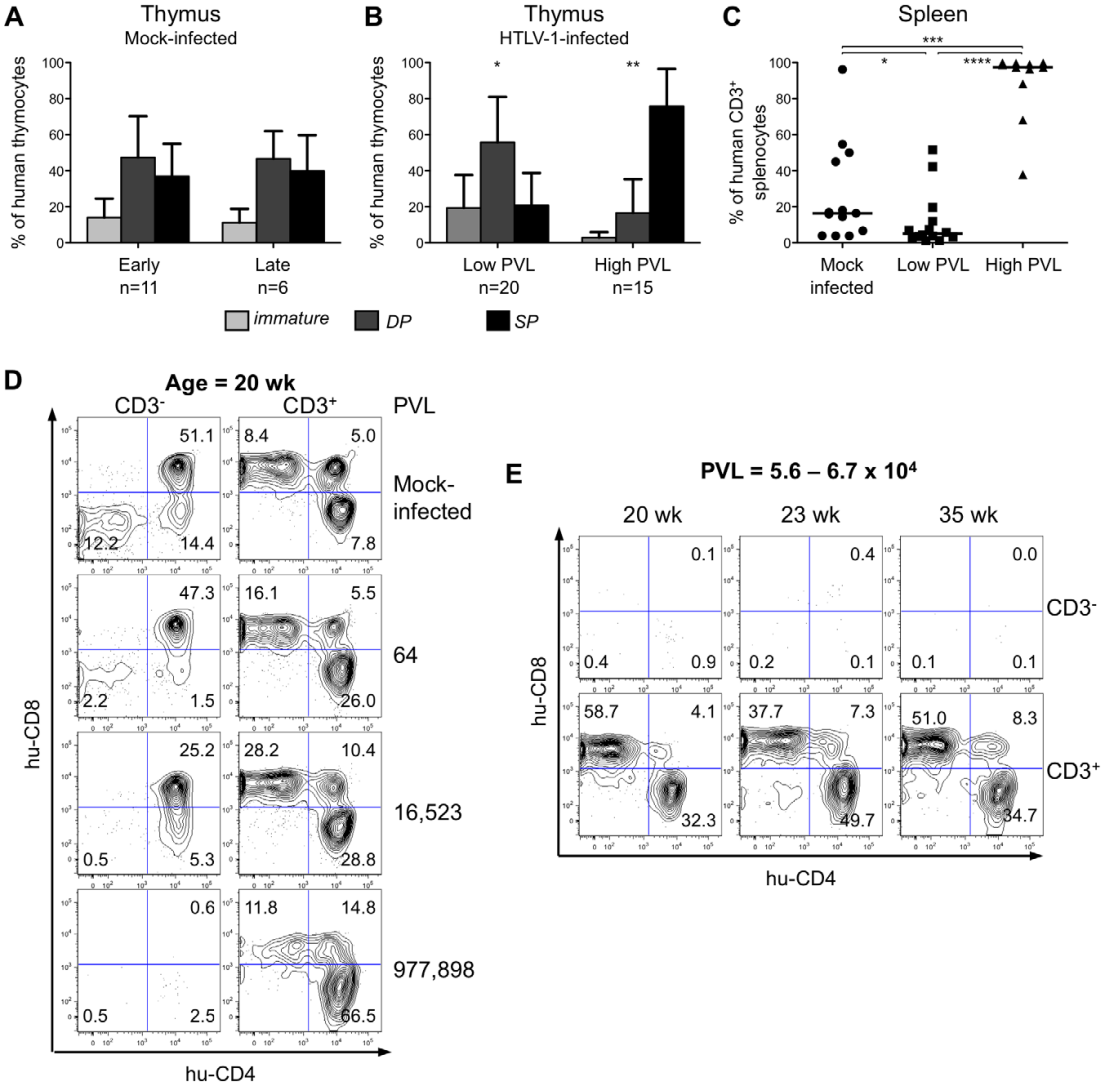
The human T-cell development is altered in HTLV-1-infected HIS Rag2^-/-^γ_c_^-/-^ mice. (**A**) Composite data from 11 mock-infected mice sacrificed early after CD34^+^ cell transplantation (early) and 6 mock-infected mice sacrificed later on (late). Bar graphs represent percent of human CD45^+^ thymocytes that are CD4^-^CD8^-^ and CD4ISP immature cells (light grey), DP cells (dark grey) and SP cells (black). The graphs present the mean and the SD. Statistical differences were calculated using the χ2 test: *P<0.05, **P<0.01. (**B**) Composite data from 20 HTLV-1-infected mice with a low PVL and 15 HTLV-1-infected mice with a high PVL showing a decrease in the frequency of the immature thymocytes (light grey) concomitant with an increase of that of the mature SP cells (black) in high PVL mice. (**C**) Expansion of human CD3^+^ T-cells at the periphery after HTLV-1 infection: FACS analysis of CD3 expression by hCD45^+^ splenocytes in 35 HIS Rag2^-/-^γ_c_^-/-^ either mock- or HTLV-1-infected mice. The median values are indicated by horizontal lines. Statistical differences were calculated using the Mann-Whitney's U test: *P<0.05, ***P<0.001, ****P<0.0001. (**D**) Correlation between HTLV-1 proviral load, the depletion of immature thymocytes and the expansion of mature thymocytes. FACS plots of the immature CD3^-^ subpopulations containing DN (CD4^-^CD8^-^), immature DP and CD4ISP and the mature CD3^+^ subpopulations, stained for human CD4 and CD8 are shown for representative animals either mock-infected or HTLV-1-infected with different PVL (indicated on the right side) and killed at 20 weeks after CD34^+^ cell transplantation. (**E**) The alteration of the human thymopoiesis induced by HTLV-1 is independent on the duration of the infection period: FACS analysis of the human CD3^-^ and CD3^+^ thymocyte subpopulations in three HTLV-1-infected mice sacrificed at different weeks after CD34^+^ cell transplantation, but each with a similar high PVL (from 5.6×10^4^ to 6.7×10^4^ copies/10^5^ cells).

To investigate the effect of HTLV-1 infection on αβT cell development in HIS Rag2^-/-^γ_c_^-/-^ mice, we proceeded to a comparative FACS analysis of thymopoiesis in infected or in mock-infected mice. In the latter, at either 8 to 20 weeks (early) or 18 to 35 weeks (late) after PBS injection, the relative frequency of the three main human subpopulations (immature, DP and SP) remained mostly unchanged (Figure 4A). In the low-PVL HTLV-1-infected HIS Rag2^-/-^γ_c_^-/-^ mice, the respective percentages of these subpopulations were slightly different with a higher frequency of DP cells and a lower frequency of SP cells (Figure 4B). In contrast, in the high-PVL mice, a significant depletion of the immature DN subpopulation was observed together with a strong decrease of DP thymocytes and with a large increase of the mature SP cells. Such alterations were evident in mice as young as 18 weeks, *i.e.* 13 weeks after inoculation with MT2 cells. These data show that the main impact of HTLV-1 infection was on the population of the most immature T cells, as indicated by their significant decrease in mice with the highest PVL. In the spleen of infected mice, the population of mature T cells increased with the PVL level to the point where mature T cells were the only human population observed in that organ (Figure 4C).

These observations suggested a positive correlation between the PVL and the alterations of human T cell development observed in HTLV-1-infected HIS Rag2^-/-^γ_c_^-/-^mice. To verify this suggestion, we analysed CD4 and CD8 expression on thymocytes of either the less mature CD3^-^ or the more mature CD3^+^ subpopulations of four age-matched mice including one mock-infected mouse and three HTLV-1-infected mice, one with a low PVL and two with high PVL (Figure 4D). We observed a significant decrease in the CD3^-^ subset, which correlated with the increase in PVL. In fact, the immature DN, CD4ISP and DP subpopulations were severely depleted in the mouse with the highest PVL (left panel), whereas the CD3^+^ SP cells increased to 78.3% in the same mouse (right panel). The thymocyte subpopulations in three mice with a comparable high PVL, but killed at different times after infection (20, 23 and 35 weeks) displayed a similar profile, *i.e.* depletion of CD3^-^ immature and DP cells and increase of CD3^+^ mature SP cells (Figure 4E). These results show that HTLV-1 infection led to an increased number of mature T cells in the thymus and in the periphery. We conclude that HTLV-1 infection perturbed the development of αβT lymphocytes in the thymus of HIS Rag2^-/-^γ_c_^-/-^ mice, mainly by propelling immature thymocytes towards the late stages of maturation. We now propose to investigate whether such an effect is induced by the viral regulatory Tax.

### Activation of survival and proliferating pathways in HTLV-1-infected HIS Rag2^-/-^γ_c_^-/-^ mice

**Figure 5:**
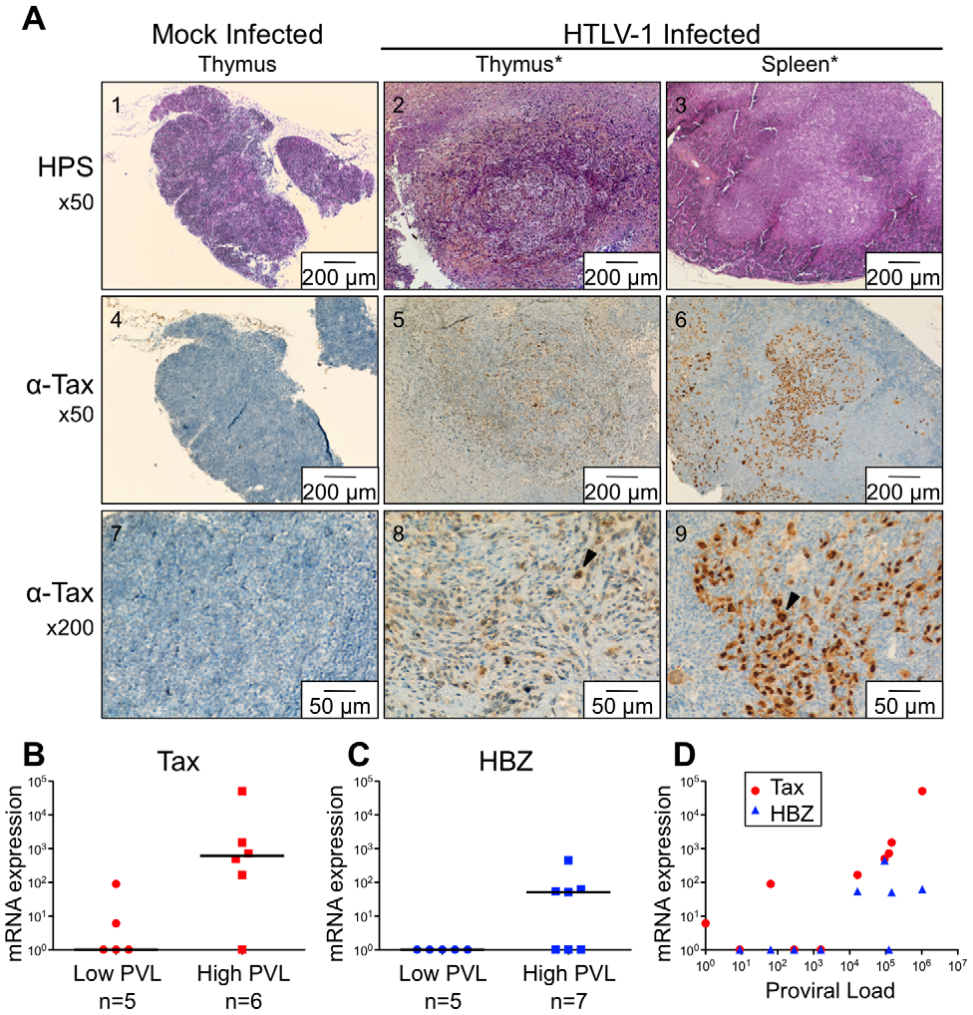
Expression of Tax and HBZ in thymocytes isolated from HTLV-1-infected HIS Rag2^-/-^γ_c_^-/-^ mice. (**A**) Immunohistological characterization of representative sections of thymus and spleen from HTLV-1- and mock-infected HIS mice. The thymus of control mice (1) shows a normal architecture, whereas that of HTLV-1-infected mice contains a dense cellular infiltrate made of large lymphoid cells interspersed with giant multinucleated cells (2). A disorganized architecture is also observed in the infected spleen; the white pulp is hyperplastic and is made of large lymphoid aggregates containing large lymphoid cells and multinucleate cells; the red pulp shows extramedullary hematopoiesis with myeloid and erythroid elements (3). Tax immunostaining reveals that the thymus and spleen of infected animals displayed large lymphoma cells with a nuclear localization of Tax (5,6,8,9). Infiltration of lymphomatous cells expressing Tax was not observed in control mice (4,7). (**B-C**) Tax and HBZ mRNA loads in thymocytes isolated from HTLV-1-infected HIS Rag2^-/-^γ_c_^-/-^ mice. Total RNAs were extracted from thymocytes of infected mice with either a low or a high PVL, and levels of mRNA coding for Tax and HBZ were measured by RT-qPCR and normalized to b-actin. The zero value of Tax and HBZ gene transcripts was observed in 60 and 100% of mice with low PVL, respectively; the median values are indicated by horizontal lines. (**D**) Dot plot graph of Tax and HBZ mRNA loads as a function of the proviral load.

Of the 35 HIS Rag2^-/-^γ_c_^-/-^ mice that were inoculated with HTLV-1-producing MT2 cells, 13 mice displayed pathological features, the severity of which appeared to correlate with proviral load and with the number of activated CD25^+^ T-cells in the thymus (Table 1). These pathological features were observed only in high-PVL mice that were killed between 16 and 35 weeks (Figure 5A). While most of these mice only displayed enlarged lymph nodes, some of them also developed hepatosplenomegaly, lymphadenopathy, and thymoma (compare panel 2 to panel 1) in which the cells were found to express the Tax protein detected by immunohistochemical analysis (panels 5,6,8,9).

**Table 1:** Pathological features in HIS Rag2^-/-^γ_c_^-/-^ mice infected with HTLV-1.

Mouse*	Age of Infection	Age of Analysis	Proviral Load °	observations
#209	7 wk	28 wk	1.58×10^3^	
#178	5 wk	18 wk	5.39×10^3^	Enlarged LN
#365	5 wk	21 wk	1.05×10^4^	Enlarged LN
#273	4 wk	20 wk	1.65×10^4^	Enlarged LN DP T cells in blood, spleen and Bone marrow
#189	4 wk	16 wk	3.42×10^4^	Enlarged LN
#297	7 wk	32 wk	4.75×10^4^	
#357	5 wk	23 wk	7.30×10^4^	Enlarged LN Tumor in the liver Leukemia
#248	7 wk	20 wk	8.12×10^4^	
#240	7 wk	23 wk	9.37×10^4^	Enlarged LN Tumor in the liver and the spleen
#307	7 wk	32 wk	9.74×10^4^	
#242	7 wk	20 wk	1.15×10^5^	Enlarged LN
#279	7 wk	35 wk	1.23×10^5^	Enlarged LN Thymoma
#200	8 wk	29 wk	1.46×10^5^	
#278	7 wk	33 wk	2.12×10^5^	Enlarged LN Tumor in the spleen
#190	4 wk	18 wk	9.78×10^5^	Thymoma
#188	4 wk	30 wk	1.04×10^6^	Enlarged LN Thymoma

*Mice were inoculated intraperitoneally with irradiated MT2 cells.

° Proviral Load is expressed as number of proviral copies per 10^5^ thymocytes.

Based on our previous experiments on human immature thymocytes, these *in vivo* observations therefore suggested that Tax induced the alterations in thymopoiesis observed in HIS Rag2^-/-^γ_c_^-/-^ mice. *tax* transcription was readily detected in the thymus of high-PVL mice, whereas in the low-PVL mice, it was very low or undetectable (Figure 5B). *hbz* transcription displayed the same pattern (Figure 5C). Finally, the observed increase in Tax transcription correlated with proviral load, unlike HBZ transcripts (Figure 5D).

**Figure 6:**
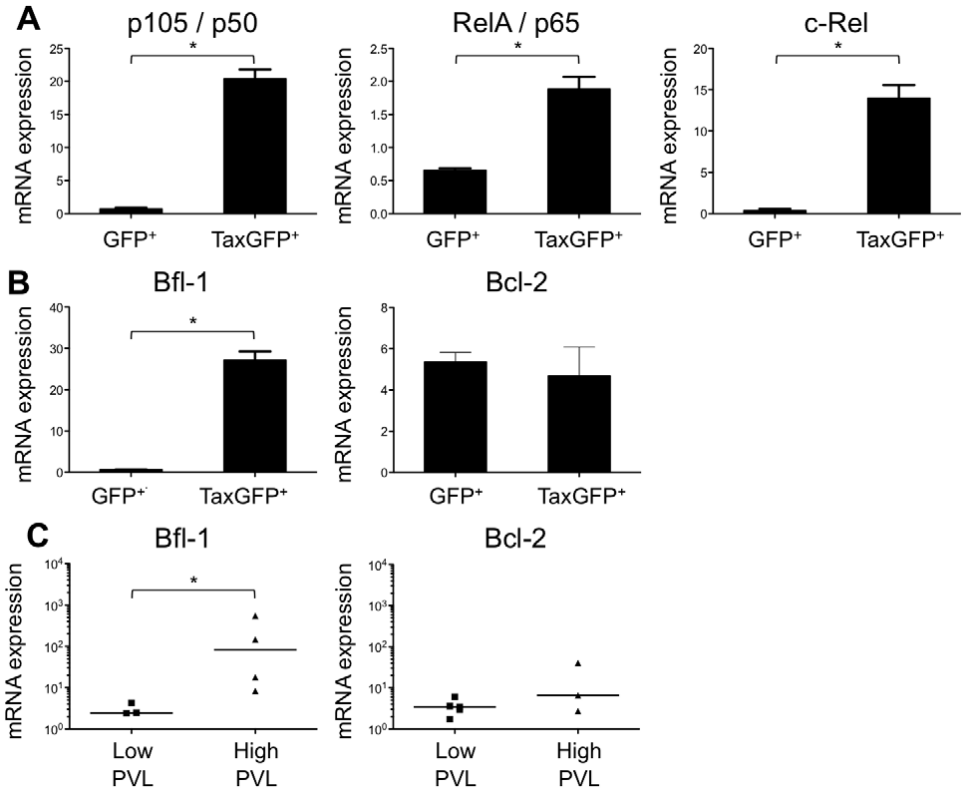
Enhanced transcription of the NF-κB and the antiapoptotic *Bfl1* genes by Tax. (**A-B**) Immature thymocytes isolated as previously described [12] were nucleofected with either pCMV-TaxGFP or pCMV-GFP. Twenty-four hours later, GFP^+^ cells were sorted, and the total RNAs were isolated and reverse transcribed. The cDNA samples were subjected to qPCR using primers specific for the indicated genes and normalized for the amount of cDNA, using human β-actin as an internal control. Standard deviations are from at least two determinations performed in triplicate. * P<0.05 by Mann-Whitney U test. (**C**) *In vivo* correlation between the *Bfl1* gene transcription and the HTLV-1 proviral load: total RNAs were extracted from thymocytes of HTLV-1-infected HIS Rag2^-/-^γ_c_^-/-^ mice from each group, and levels of *bfl-1* and *bcl-2* mRNAs normalized to human b-actin were measured by RT-qPCR. Levels of *bfl-1* mRNAs were significantly higher in high PVL mice than in low PVL mice, whereas *bcl-2* transcription was unchanged. The median values are indicated by horizontal lines. * P<0.05 using the Mann-Whitney U test.

We previously reported that Tax protein interferes with assembly of the pre-TCR when expressed *in vitro* in human immature thymocytes, such as the CD4ISP thymocytes [12], [13]. During normal αβT-cell development, signals from the pre-TCR activate the NF-κB pathways that control the expression of target genes implicated in cell proliferation and survival [17]. We hypothesized that Tax might compensate for the absence of a functional pre-TCR by inducing NF-κB activation in immature thymocytes. We therefore investigated the impact of Tax overexpression on the transcription of NF-κB-related genes (RelA/p65, p105/p50, c-Rel) in immature thymocytes. We observed that the amount of the respective transcripts was much greater in TaxGFP-expressing immature thymocytes than in GFP^+^ control cells (Figure 6A). We also analysed isolated mRNAs to detect transcripts of the anti-apoptotic genes *Bfl-1* and *Bcl-2*, because *Bfl-1* is a target of NF-κB transcriptional activity whereas Bcl-2 is independent of NF-κB. Only the expression of *Bfl-1* was significantly increased in Tax-expressing thymocytes (Figure 6B). These results show that Tax expressed in human immature thymocytes activates the transcription of NF-κB factors and that of the Bfl-1 anti-apoptotic gene.

**Figure 7:**
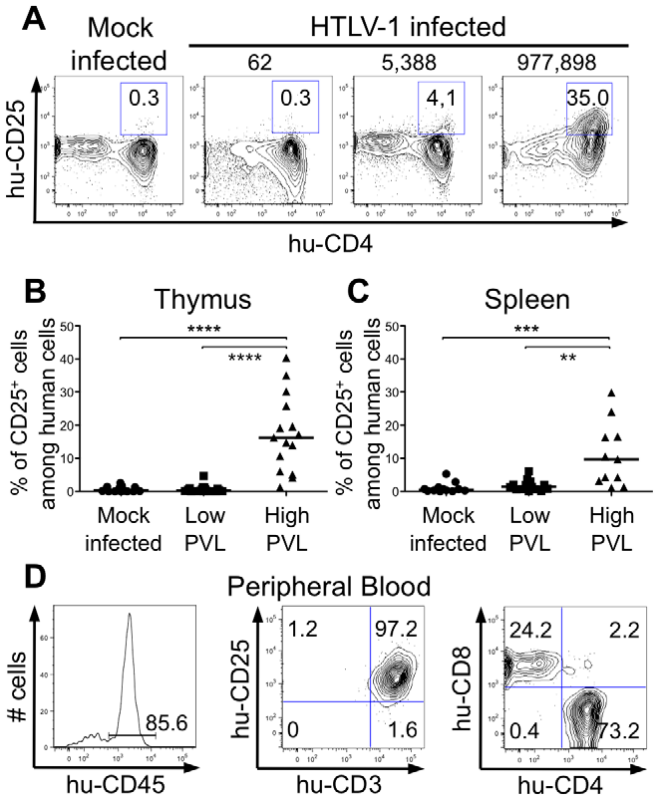
Correlation between expression of CD25 activation marker and HTLV-1 proviral load. (**A**) Representative flow cytometry plots showing the expression of human CD25 and CD4 markers on thymocytes of 17 mock- and 35 HTLV-1-infected HIS Rag2^-/-^γ_c_^-/-^ mice with different PVL (indicated on top). (**B**) Frequency of human CD4^+^ cells expressing CD25 among human thymocytes of 35 HIS Rag2^-/-^γ_c_^-/-^ HTLV-1-infected mice; the median values are indicated by horizontal lines. (**C**) Frequency of human CD4^+^ cells expressing CD25 among human splenocytes of 35 HIS Rag2^-/-^γ_c_^-/-^ mice; the median values are indicated by horizontal lines. Statistical differences were calculated using the Mann-Whitney U test: **P<0.01, ***P<0.001, ****P<0.0001. (**D**) Lymphoproliferation in the peripheral blood of one HTLV-1-infected HIS Rag2^-/-^γ_c_^-/-^ mouse with a high PVL (7.3×10^4^ copies/10^5^ cells): this flow cytometry analysis shows that among the 85.6% of huCD45+ cells (left panel), 97.2% are activated T lymphocytes (center panel), the majority with a CD4^+^ phenotype (right panel).

We therefore investigated whether the NF-κB dependent expression of these two genes, *bcl2* and *bfl1*, was increased in HTLV-1-infected HIS Rag2^-/-^γ_c_^-/-^ mice. RT-qPCR analysis showed a clear increase in the transcription of *Bfl-1* in these mice, when compared to control animals (Figure 6C); this increase was comparable to that found in Tax-expressing thymocytes (Figure 6B). The increase in *Bfl-1* mRNA correlated with that of *tax* mRNA, as did CD25 mRNA, which is also dependent on NF-κB factors. Indeed, a comparative FACS analysis of mock-infected and HTLV-1 infected HIS Rag2^-/-^γ_c_^-/-^ mice indicated that the number of hu-CD45^+^ CD25^+^ cells in the thymus and in the spleen cells was significantly higher in high PVL mice than in low PVL or in control mice (Figure 7A, B, C). Lastly, activated T lymphocytes, which were mainly CD4^+^, were also found in the peripheral blood of an HTLV-1-infected HIS Rag2^-/-^γ_c_^-/-^ mouse (Figure 7D). This finding demonstrates that HTLV-1 infection induced expansion of mature T-cells in the periphery. We conclude that Tax activates the transcription of genes involved in cell survival and proliferation in the thymus of HTLV-1–infected HIS Rag2^-/-^γ_c_^-/-^ mice and favours the expansion of mature activated T cells. Thus, the presence of T-cell proliferation in HTLV-1 infected HIS Rag2^-/-^γ_c_^-/-^ mice indicates that HTLV-1 infection of the thymus might be a critical pre-leukemogenic event.

## Discussion

During the last decade, humanized mouse technology has made remarkably rapid progress, allowing the establishment of high levels of human chimerism in various host organ/tissues, particularly the immune system, liver and muscle [14], [21]–[23]. These humanized mice appear ideally suited for direct investigation of human infectious agents [24], [25]. In the present study, we have used Rag2^-/-^γ_c_^-/-^ mice, which recapitulate the main events in the development of the human lymphoid compartment [26]–[28]. These mice have already displayed interesting potential for studying infection with lymphotropic viruses, such as HIV (Human Immunodeficiency Virus) and EBV (Epstein-Barr Virus) [29]–[34].

In the present study, we used HIS Rag2^-/-^γ_c_^-/-^ mice inoculated with irradiated HTLV-1-producing cells, at a time when the human lymphoid compartment has been established, *i.e.* within a period of 1 to 2 months after transplanting BALB/c Rag2^-/-^γ_c_^-/-^ immunodeficient animals with human CD34^+^ cord blood cells. This infection protocol was found to be very efficient, as in most of the inoculated HIS Rag2^-/-^γ_c_^-/-^ mice the provirus was found integrated in the genome of human cells. These mice were analyzed at regular intervals within a 7-month period after inoculation. We observe a sequential increase of the proviral load, which correlated with the appearance of critical alterations in the distribution of T-cell subsets in the chimeric thymus. Specifically, we observed slight differences in the respective percentages of the three main thymocyte subsets (immature, DP and SP) between HIS Rag2^-/-^γ_c_^-/-^ mice with a low proviral load and mock-infected animals. Conversely, in mice with a high proviral load, the SP cells predominated at the expense of the immature and DP populations. These data provide strong evidence that *in vivo* HTLV-1 infection of HIS Rag2^-/-^γ_c_^-/-^ mice perturbs human T-cell development in the thymus at the level of immature cells, by propelling development towards the mature stages. In addition, the infected mice had a high proviral load in the same range as that found in ATL patients [19]. These findings demonstrate that HIS Rag2^-/-^γ_c_^-/-^ mice infected with HTLV-1 provide a suitable model for viral-induced malignancy. They further suggest that the combination of immature target cells in the thymus and the immunodeficient environment of HIS Rag2^-/-^γ_c_^-/-^ mice favours the rapid development of a T-cell malignancy.

This model will also be helpful for studying the involvement of other HTLV-1 genes like *hbz* in viral pathogenesis as well as the role of the proviral integration sites. Thus, an oligoclonal expansion of HTLV-1-infected cells was observed in the periphery of five infected HIS Rag2^-/-^γ_c_^-/-^ mice. In contrast, preliminary results obtained from one HTLV-1 infected mouse displaying a thymoma indicate an extraordinary degree of polyclonality of integration sites in the thymus (Figure S1A). Interestingly, the three clones that dominated the population in both spleen and lymph nodes were not dominant in the thymus, and the three most abundant clones observed in the thymus were not detected in the periphery (Figure S1A, B, C).

Our observations are consistent with those reported by Banerjee *et al.* who used a different infection strategy as well as different immunodeficient mice [10]. Indeed, these authors generated HTLV-1-infected humanized nonobese diabetic severe combined immunodeficiency (HU-NOD/SCID) mice by inoculating 4- to- 6-week NOD/SCID mice with human fetal liver-derived CD34^+^ cells infected *ex vivo* with HTLV-1. Less than half of these infected mice developed T-cell lymphomas with a CD4^+^CD25^low^ or CD4^+^CD25^low^ phenotype. These mice also showed greater proliferation of infected human CD34^+^ cells in the bone marrow, leading the authors to propose that HTLV-1 can transform CD34^+^ cells. However, HTLV-1 proviral sequences were not detected in CD34^+^ bone marrow cells from ATL patients [35]. Banerjee *et al.* also reported that HTLV-1 infection skewed T-cell development in the thymus of these HTLV-1-infected HU-NOD/SCID mice, as well as in the thymus of HTLV-1-infected HU-NSG (NOD/SCID IL-2γ chain^null^) mice. These data support our present observations which suggest that alterations of T-cell development play a prominent role in the initiation of HTLV-1-induced leukemogenesis.

In a previous study, we demonstrated that the viral Tax regulatory protein can inactivate the activity of bHLH E2A transcription factors [12]. This inhibitory effect leads to a decrease of the transcription of the pTα gene in immature thymocytes, thus precluding the assembly of the pre-TCR and interfering with the β-selection checkpoint [13]. The pre-TCR plays a crucial role in T-cell development, by allowing the maturation of thymocytes with a functional β chain. Signalling from the pre-TCR in the β-selected cells leads to the activation of NF-κB, which provides survival and proliferative advantages [18], [36]. We hypothesize that Tax, which is able to activate the NF-κB pathways, compensates for the absence of the pre-TCR in HTLV-1-infected immature thymocytes. Thus, Tax-activated NF-κB would prevent apoptosis and drive proliferation. We indeed observed that activated transcription of the NF-κB pathways in *ex vivo* Tax-expressing immature thymocytes correlated with increased transcription of *Bfl1*, an anti-apoptotic NF-κB-responsive gene. Likewise, we found increased transcription of Bfl-1 in thymocytes of infected mice. We also observed an expansion of mature SP cells in the thymus and in the spleen of HTLV-1-infected HIS Rag2^-/-^γ_c_^-/-^, and an increase in the percentage of CD25^+^ CD4^+^ T-cells. Finally, pathological features observed in some of these mice might be related to the disturbances of the αβT-cell development induced by HTLV-1 infection.

We conclude that HTLV-1 infection can override the pre-TCR checkpoint by forcing immature thymocytes to progress towards a more differentiated phenotype (acquisition of CD4 and CD8) in the absence of the pre-TCR. The Tax protein may play a key part in this process. The observed disturbances of thymopoiesis caused by HTLV-1 infection may represent critical events in the initiation of the leukemogenic process associated with this retroviral infection. More specifically, they underline the relationships between dysregulated T-cell development and neoplastic transformation and provide another contribution to the identification of molecular and cellular mechanisms involved in leukaemogenesis [37]. Indeed, the constitutive expression of Tax in immature thymocytes at the level of the β-selection checkpoint alters the developmentally regulated interplay between pre-TCR signalling and E2A activities, resulting in leukemia/lymphoma promotion.

HTLV-1-associated T-cell leukaemia strongly resembles the Notch3-induced T-cell malignancies in several respects [38]. Notch3, a member of the Notch family, is known to play a critical role in T-cell development and its constitutive activation has been related to T-cell leukaemia in both animal models and human diseases. The development of the T-cell leukaemogenic process requires the combined expression of Notch3 and the pre-TCR linked to a sustained down-regulation of E2A activity [37]. It is noteworthy that in the absence of the pre-TCR, Notch3, like Tax, can force immature T-cells through otherwise pre-TCR-dependent developmental stages and favour the progression toward a more differentiated phenotype by acquisition of CD4 and CD8. In contrast with Notch3, Tax is able to activate both the alternative and canonical NF-κB pathways, implying that the appearance of lymphoid tumors in HTLV-1 infected is linked to the exclusive activity of Tax. However, even if Tax down-modulates the transcription of pTα, leukemogenesis may also require continued weak expression of the pre-TCR, which cooperates with pTa to reach a critical level of NF-κB activation. Future experiments should be performed to verify that hypothesis.

In conclusion, our study demonstrates that BALB/c Rag2^-/-^γ_c_^-/-^ mice transplanted with human CD34^+^ cord blood cells provides an *in vivo* model to define the early steps of HTLV-1 infection that might lead to a better understanding of the initiation of the HTLV-1 associated leukemia. Our observations provide evidence that HTLV-1 infection in the thymus propels infected thymocytes toward the mature stages of T-cell development and favours the activation and the proliferation of these cells, thus providing a substrate population for further altered gene expression that would allow the emergence of a malignant clone.

## Materials and Methods

### Ethics statement

Umbilical cord blood was obtained from healthy full-term newborns with written parental informed consent according to the guidelines of the medical and ethical committees of Hospices Civils de Lyon and of Agence de Biomédecine, Paris, France. Experiments using cord blood were approved by both committees and were performed in full compliance with French law.

Animal experimentation was performed in strict accordance with the French “Comité National de Réflexion Ethique sur l’Expérimentation Animale, n°15” and the ethical guidelines for the care of these mice of the Plateau de Biologie Experimentale de la Souris (PBES, UMS 3444) at Ecole Normale Supérieure de Lyon. The protocol used throughout that study was approved by the Committee on the Ethics of Animal Experiments of the Ecole Normale Supérieure de Lyon (Permit number: 0231). All efforts were made to minimize animal suffering.

### Isolation of human CD34^+^ cells from cord blood samples

After density gradient centrifugation of human cord blood, CD34^+^ cells were enriched twice using immunomagnetic beads according to the manufacturer instructions (CD34^+^ MicroBead Kit, Miltenyi Biotec, Bergisch-Gladbach, Germany). Purity (≥95%) was evaluated by FACS analysis using human CD34, CD3 and CD19 antibodies. Cells were either frozen or immediately transplanted when newborn mice were available.

### Generation of HIS Rag2^-/-^γ_c_^-/-^ mice

Rag2^-/-^γ_c_^-/-^ mice on a BALB/c background were bred and maintained under specific pathogen-free conditions in accordance with the French law and the guidelines of PBES. Rag2^-/-^γ_c_^-/-^ mice were transplanted with human CD34^+^ cells, as previously described [16], [39]. Briefly, newborn (less than 3-day old) mice were irradiated twice within a 3-hour interval with 2×2 Gy from a ^137^Cs source (CIS BIO international, IBL 637) at 1.28 Gy/min., a dose that was adjusted to be sub-lethal. After the second irradiation, mice were injected intrahepatically with 2×10^5^ human CD34^+^ cord blood cells in 30 µl PBS.

### HTLV-1 infection of HIS Rag2^-/-^γ_c_^-/-^ mice

The HTLV-1 T-cell line MT2 [40] was irradiated with 77 Gy from a ^137^Cs source (CIS BIO international, IBL 637) at 1.28 Gy/min. This dose was adjusted to inhibit cell proliferation but not HTLV-1 production. HIS Rag2^-/-^γ_c_^-/-^ mice aged from 4 to 8 weeks were intraperitoneally injected with 2×10^6^ lethally irradiated MT2 cells in 100 µl PBS. Mock-infected mice were injected with PBS alone. Mice were sacrificed at different time-points after infection. Infection was performed in a Biosafety Level 3 laboratory in accordance with the PBES guidelines.

### Histopathological analysis

Anesthetized mice were killed and tissue specimens (thymus, spleen, bone marrow and lymph nodes) were fixed directly in neutral buffered formaldehyde, embedded in paraffin, sectioned and stained with H&E solution. An indirect immunoperoxidase technique was applied to 4 µm-thick formalin-fixed, paraffin-embedded tissue sections with a rabbit polyclonal anti-Tax antibody. An automated immunostainer was used (Ventana, Tucson, AZ, USA). Antigen unmasking and immunodetection were performed according to the manufacturer's instructions (Ventana). The avidin-biotin technique was used for development.

### Flow cytometric analysis

Peripheral blood cells were collected from the retro-orbital venous sinus under Ketamine-Xylazyne anesthesia. When needed, mice were sacrificed after anesthesia and thymus, spleen, bone marrow and lymph nodes were collected, gently minced in PBS to obtain a single-cell suspension. For FACS analysis, monoclonal antibodies, conjugated with either FITC, PE, PE-Cy7, APC or V450 against the following human antigens were used: CD4 (RPA-T4), CD8 (RPA-T8), CD19 (HIB19), CD34 (581/CD34), CD45 (HI30), (all BD Biosciences, San Diego, CA), CD3 (UCHT1) and CD25 (BC96) (eBioscience, San Diego, USA). Flow cytometric analysis was performed using a FACScanto II and Diva for acquisition (Becton Dickinson Immunocytometry Systems, Mountain View, CA) and FlowJo (Treestar, Ashland, OR) for data analysis.

### DNA and RNA isolation

Genomic DNA was extracted from the single cell suspension using the Nucleospin Blood kit (Macherey-Nagel, Düren, Germany) according to the manufacturer's instructions, and resuspended in 60 µl of the supplied elution buffer. For mouse tissues, RNA was extracted from the single cell suspension using the Nucleospin RNA XS kit (Macherey-Nagel, Düren, Germany) according to the manufacturer's instructions, and resuspended in 10 µl of water. For human thymus, total RNA was extracted from 1–2×10^5^ thymocytes using TRIzol reagent (Invitrogen) according to the manufacturer's instructions. Two chloroform extractions were performed on the organic phase to avoid phenol contamination. Before reverse transcription, RNAs were first treated with 10 U of RNase-free DNase I (Qiagen) for 20 min at 27°C and then for 15 min at 60°C.

### PCR, RT-PCR and quantitative real-time PCR (qPCR)

The RNA samples were reverse transcribed at 42°C for 1 hour in a total volume of 20 µl reaction buffer containing 100 U of SuperScriptTII Rnase H- reverse transcriptase (RT; Invitrogen). A reaction without RT was performed in parallel to serve as control for the absence of DNA contamination. PCR was performed using the Phusion enzyme (Finnzyme, Espoo, Finland) in a Piko thermocycler (Finnzyme, Espoo, Finland). For ALU-PCR, the first round was carried out using an ALU-specific primer and a *gag* specific primer (see sequence below). The amplification product was then diluted in water (1/50) and used as templates for a *gag*-specific PCR. The control PCR for *gag* was carried out by diluting the initial template (1/1250) instead of applying first ALU-PCR.

Quantitative PCR (qPCR) was performed using the FastStart Universal SYBR Green Master (Roche, Mannheim, Germany) on a LightCycler 2.0 system (Roche Applied Science, Illinois, USA) or on a StepOnePlus system (Applied Biosystem, CA, USA).

Species-specific primers were used to amplified human β-actin, 5′–TGAGCTGCGTGTGGCTCC–3′ and 5′–GGCATGGGGGAGGGCATACC–3′, human p105/50 5′–CTGGAAGCACGAATGACAGA–3′ and 5′–CCTTCTGCTTGCAAATAGGC–3′, RelA/p65 5′–CCTGGAGCCAGGCTATCAGTC–3′ and 5′–CACTGTCACCTGGAAGCAGA–3′, c-Rel 5′-GAACGATTGGGAAGCAAAAG-3′ and 5′-GGCACAGTTTCTGGAAAAGC-3′, human Bfl-1 5′-GGCATCATTAACTGGGGAAG-3′ and 5′-TCCAGCCAGATTTAGGTTCAA-3′, human Bcl2 5′-TGTGGATGACTGAGTACCTGAACC-3′ and 5′-GTTTGGGGCAGGCATGTTGAC-3′, mouse actin 5′–TGGAATCCTGTGGCATCCATGAAAC–3′ and 5′–TAAAACGCAGCTCAGTAACAGTCCG–3′, HTLV-1 gag 5′-CCCTCCAGTTACGATTTCCA-3′ and 5′–GGCTTGGGTTTGGATGAGTA–3′ , tax 5′–GTTGTATGAGTGATTGGCGGGGTAA–3′ and 5′–TGTTTGGAGACTGTGTACAAGGCG–3′, hbz 5′-GGCAGAACGCGACTCAACCG-3′ and 5′-GCCGATCACGATGCGTTTCCC-3′; in this later case, PCR amplifications were conducted on oligo(dT)-primed cDNAs with a forward primer spanning the HBZ-SI mRNA exon 1 and a reverse primer located in the exon 2; these primers do not overlap with the splice sites, avoiding false positive. Amplification of human β-actin was done for each experimental sample to normalize results in RT-PCR experiments.

### Evaluation of the proviral load

The proviral load was expressed as number of copies of provirus per 100,000 human cells. It was evaluated by qPCR on genomic DNA for the number of copies of tax and the number of copies of human β-actin. To establish the calibration curve we assumed that one ng of DNA extracted from human PBMC contains 333 copies of β-actin. TARL-2 is a rat HTLV-1 infected cell line which has a single copy of HTLV-1 proviral DNA per cell and we therefore assume that one ng of DNA contains 167 copies of the *tax* gene [41].

### Cell transfection and plasmids

Human immature thymocytes were obtained as previously described [12], using the EasySep CD3-positive selection cocktail to deplete CD3^+^ cells followed by negative selection using the human CD4^+^ T-cell enrichment cocktail (StemCell Technologies Inc, Vancouver, BC, Canada) according to the manufacturer's instructions. The Tax expression plasmid, pCMV-TaxGFP (generous gift from R. Mahieux), contains the *tax* coding sequence in frame with the N-terminal extremity from the *gfp* sequence under the control of the cytomegalovirus promoter. pMax-GFP (Amaxa) was used as a GFP-control expressing vector. Immature thymocytes (2.10^6^ cells), cultured overnight in α-MEM complete medium containing 20 ng/ml IL-7 (R&D, Abingdon, UK) and 10 ng/ml SCF (Peprotech, Rocky hill, NJ), were transfected either with 4 µg of pCMV-TaxGFP or pCMV-GFP by nucleofection (Human CD34 cell nucleofector kit, Amaxa, Köln, Germany) according to the manufacturer's instructions. Twenty-four hours later, GFP^+^ cells were sorted from both cultures using an ARIA sorter (BD-Beckinson).

### Selective amplification and quantification of proviral insertion sites

DNA was extracted from tissue samples as described in the section “DNA and RNA isolation” and the selective amplification and quantification of the proviral insertion sites was done as previously described [20]. The DNA was sheared by sonication. A linker containing a tag was ligated, and nested PCR was performed between the end of the HTLV-1 LTR and the linker. Nested PCR products were pooled to construct the sequencing library. A paired-end read (read 1 and read 2) plus a tag index read were acquired on an Illumina Genome Analyzer II. Read 1 and read 2 were mapped against the human genome (build hg18) and the proviral insertion site and the shear site were respectively deduced. The relative abundance (in percent of proviral load) of a given unique insertion site (UIS) was calculated from the number of amplicons of different length (*i.e.* different shear sites). For more details, see [20].

### Statistical analysis

Statistical analyses were performed using the GraphPad Prism software and the Mann-Whitney U test or the χ2 test.

## Supporting Information

Figure S1 **Distribution of abundance of unique insertion sites in different tissues of the same mouse.** The HTLV-1 clonal structure in each genomic DNA sample is depicted by a pie chart. Each slice represents one unique insertion site (UIS); the size of the slice is proportional to the relative abundance of that UIS. (**A**) The 3 most abundant UISs were colored in blue. These UISs were found neither in the spleen nor in the lymph node sample. (**B, C**) The 3 most abundant UISs were colored in red/orange. 11_82937981 means that the provirus of this clonal population is inserted in chromosome 11, coordinate 82937981. These 3 major UISs were detected in the thymus but at a relatively low abundance.
(TIFF)
