# Supplementary figures and images for: HTLV-1 Propels Thymic Human T Cell Development in “Human Immune System” Rag2-/- gamma c-/- Mice
Source: PLoS Pathog. 2011 Sep 1;7(9):e1002231. doi: 10.1371/journal.ppat.1002231 (PMC3164654; doi:10.1371/journal.ppat.1002231)

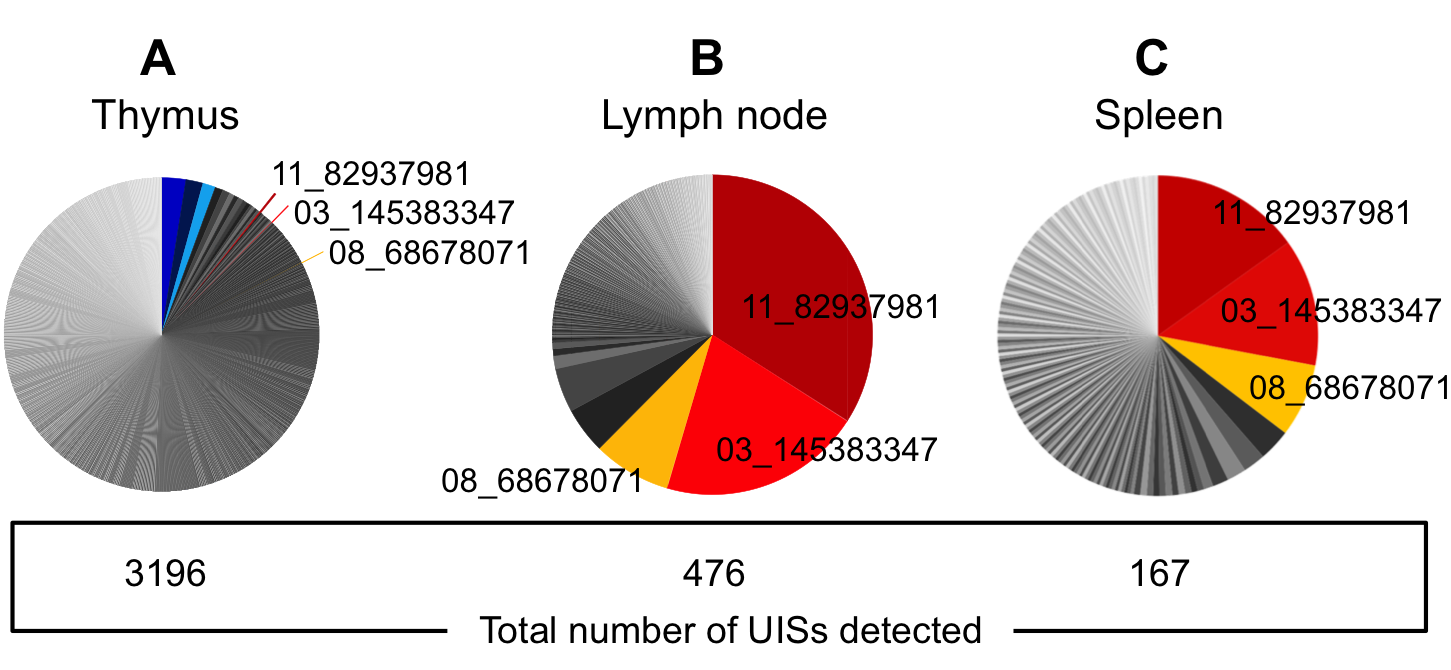

Supplement: Figure S1 — Distribution of abundance of unique insertion sites in different tissues of the same mouse. The HTLV-1 clonal structure in each genomic DNA sample is depicted by a pie chart. Each slice represents one unique insertion site (UIS); the size of the slice is proportional to the relative abundance of that UIS. (A) The 3 most abundant UISs were colored in blue. These UISs were found neither in the spleen nor in the lymph node sample. (B, C) The 3 most abundant UISs were colored in red/orange. 11_82937981 means that the provirus of this clonal population is inserted in chromosome 11, coordinate 82937981. These 3 major UISs were detected in the thymus but at a relatively low abundance. (TIFF) [file ppat.1002231.s001.tiff]
